# Synthesis and Anti-Fungal Activity of Seven Oleanolic Acid Glycosides

**DOI:** 10.3390/molecules16021113

**Published:** 2011-01-26

**Authors:** Hanqing Zhao, Guanghui Zong, Jianjun Zhang, Daoquan Wang, Xiaomei Liang

**Affiliations:** Key Lab of Pesticide Chemistry and Application Technology, Department of Applied Chemistry, China Agricultural University, Beijing 100193, China

**Keywords:** synthesis, oleanolic acid, glycoconjugate, anti-fungal activity

## Abstract

In order to develop potential anti-fungal agents, seven glycoconjugates composed of α-L-rhamnose, 6-deoxy-α-L-talose, β-D-galactose, α-D-mannose, β-D-xylose-(1→4)-6-deoxy-α-L-talose, β-D-galactose-(1→4)-α-L-rhamnose, β-D-galactose-(1→3)-β-D-xylose-(1→4)-6-deoxy-α-L-talose as the glycone and oleanolic acid as the aglycone were synthesized in an efficient and practical way using glycosyl trichloroacetimidates as donors. The structures of the new compounds were confirmed by MS, ^1^H-NMR and ^13^C- NMR. Preliminary studies based on means of mycelium growth rate, indicated that all the compounds possess certain fungicidal activity against *Sclerotinia sclerotiorum (Lib.) de Bary*, *Rhizoctonia solani Kuhn, Botrytis cinerea Pers* and *Phytophthora parasitica Dast*.

## 1. Introduction

During the course of growth and development, plants synthesize triterpenoid saponins which act as preformed chemical barriers against fungal attack [1]. Aside from their important role in plant growth, these glycosylated plant secondary metabolites show various kinds of biological activity and have been used widely as anti-inflammatory, anti-tumor, anti-HIV, and antifungal agents [2]. Consequently, triterpenoid saponin structures have become the synthetic targets of many research groups [3,4]. One common feature shared by all saponins is the presence of a sugar chain at the C-3 of the aglycone moiety [5,6]. These chains vary from saponin to saponin but usually consist of glucose, arabinose, glucuronic acid, xylose or rhamnose [7].

**Figure 1:**
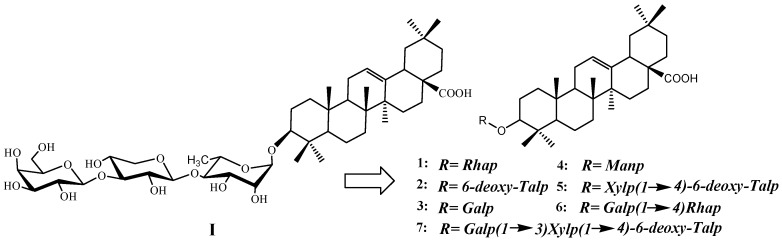
Structure of the triterpenoid saponin (**I**) and target compounds **1**–**7**.

Recently Yadava *et. al.* reported a new triterpenoid saponin isolated from the seeds of *L. scariola*, which had the structure of 3-*O*-[β-D-galactopyranosyl-(1→3)-*O*-β-D-xylopyranosyl-(1→4)-*O*-α-L-rhamnopyranosyl]-oleanolic acid (Figure 1, **I**) [8]. Interestingly, this triterpenoid saponin exhibited broad spectrum antibacterial and antifungal activities against *Staphylococcus aureus*, *Escherichia coli*, *Penicillium digitatum* and *Aspergillus niger* [8]. In a project for the discovery of novel environmentally friendly antifungal agents from natural resources, we engaged in the study of the synthesis and anti-fungal activity of glycoconjugate derivatives **1**-**7**. We report herein the preliminary results of the study.

## 2. Results and Discussion

### 2.1. Chemistry

**Figure 2:**
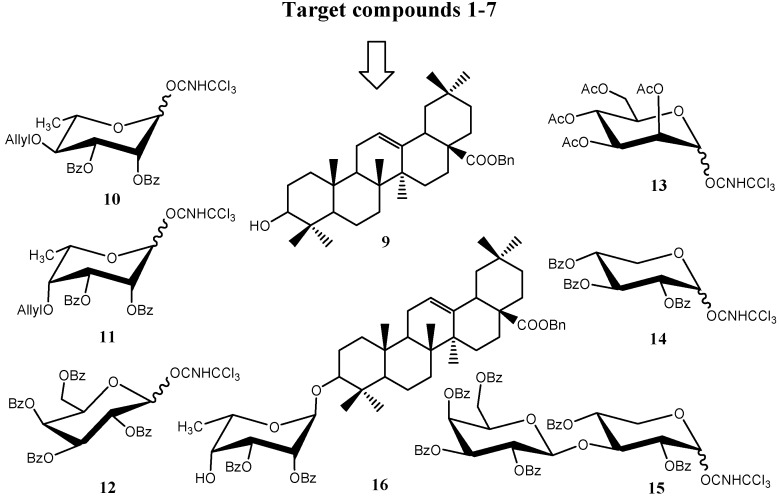
The building blocks **9-16** used for the synthesis of target compounds **1-7**.

As shown in Figure 2, we envisioned that the target compounds **1-7** could be synthesized using nine suitably protected building blocks **9**-**16**. 

**Scheme 1:**
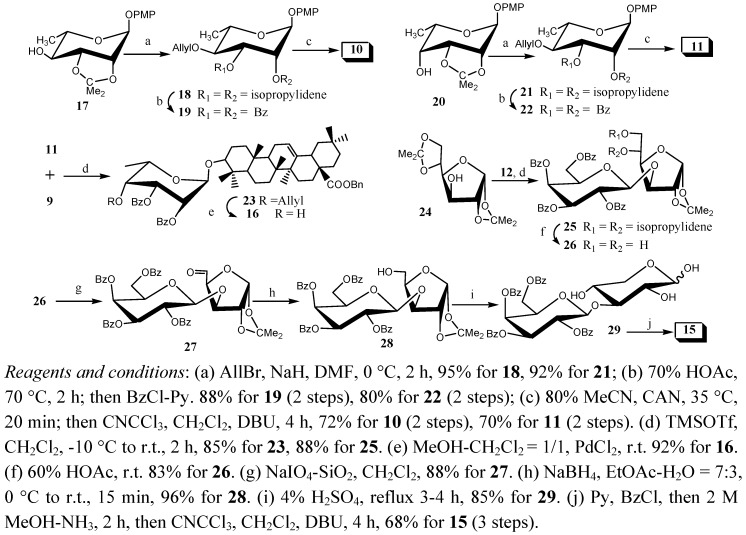
Synthetic routes to the compounds **10**, **11**, **15** and **16**.

In our work, the Schmidt method [9] was used in the glycosylation, and benzyl was chosen as the protective group for the COOH group to avoid difficulties in the final deprotection.Since the synthons **9 [10]**, **12 [11]**, **13 [12]** and **14 [13]** were easily prepared according to the reported procedures, our attention was focused on the synthesis of 4-*O*-allyl-2,3-di-*O*-benzoyl-α-L-rhamnopyranosyl trichloroacetimidate (**10**), 4-*O*-allyl-2,3-di-*O*-benzoyl-6-deoxy-α-L-talopyranosyl trichloroacetimidate (**11**), 2,3,4,6-tetra–*O*-benzoyl-β-D-galactopyranose-(1→3)-2,4-di-*O*-benzoyl-β-D-xylopyranosyl tri-chloroacetimidate (**15**)and benzyl oleanolate 3-*O*-2,3-di-*O*-benzoyl-α-L-talopyranoside (**16**) (Scheme 1). 

Among these compounds, donor **10** was prepared from the known *p*-methoxyphenyl 2,3-*O*-isopropylidene-α-L-rhamnopyranoside (**17**) [14]. Allylation of **17** with allyl bromide provided the corresponding *p*-methoxyphenyl 4-*O*-allyl-2,3-*O*-isopropylidene-α-L-rhamnopyranoside **18** quantitatively; then removal of the isopropylidene group with 70% HOAc followed by benzoylation gave **19** in high yield (88%); finally, cleavage of the *p*-methoxyphenyl glycoside in **19** with ceric ammonium nitrate (CAN) followed by trichloroacetimidation afforded the corresponding glycosyl donor **10 **in 72% yield. Meanwhile, the donor **11** was prepared in a similar way, *i.e.,* allylation of **20** [15] provided the corresponding *p*-methoxyphenyl 4-*O*-allyl-2,3-*O*-isopropylidene-6-deoxy-α-L-talo-pyranoside **21** quantitatively; and removal of the isopropylidenyl group followed by benzoylation gave **22** in high yield (80%); finally, cleavage of the *p*-methoxyphenyl glycoside in **22** with ceric ammonium nitrate (CAN) followed by trichloroacetimidation afforded the corresponding glycosyl donor **11** in 70% yield. Condensation of donor **11** with C-3-OH acceptor **9 [10]** in the presence of TMSOTf gave the α-linked 6-deoxy-taloside **23**, whose ^1^H-NMR spectrum showed characteristic signals of a doublet at *δ* 5.42 ppm (*J*_1,2_ = 3.6 Hz) for the H-1 of 6-deoxytalose, a multiplet at *δ* 5.88 ppm for CH_2_=CH-CH_2_O, and seven singlets at *δ* 1.12, 1.01, 0.92, 0.91, 0.89, 0.83, 0.60 ppm for the CH_3_ groups of oleanolic acid. Deallylation of **23** with PdCl_2_ gave the desired acceptor **16**, and the ^1^H- NMR showed that the characteristic allyl signals had disappeared.

**Scheme 2:**
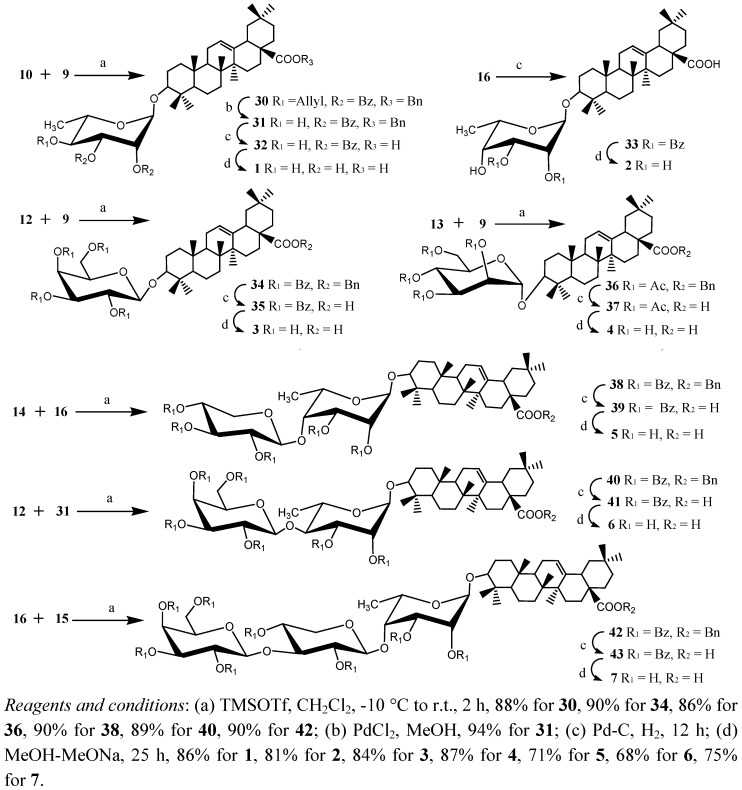
Synthesis of the target compounds **1-7**.

On the other hand, we have also developed a novel strategy for the synthesis of 2,3,4,6-tetra–*O*-benzoyl-β-D-galactopyranose-(1→3)-2,4-di-*O*-benzoyl-β-D-xylopyranosyl trichloroacetimidate**15** from 2,3,4,6-tetra–*O*-benzoyl-β-D-galactopyranosyl trichloroacetimidate **12 **and 1,2:5,6-di-*O*-isopropylidene-α-D-glucofuranose **24 **[16]. 2,3,4,6-Tetra-*O*-benzoyl-β-D-galactopyranose-(1→3)-1,2-*O*-isopropylidene-β-D-xylose **28** was conveniently prepared from **25** in 70% overall yield, via selective removal of the 5,6-*O*-isopropylidene group followed by NaIO_4_ oxidation and NaBH_4_ reduction in a similar way as reported in [17]. Subsequently, hydrolysis of **28 **was carried out in an aqueous solution of sulfuric acid (4%) under heating at reflux, and the reaction was accompanied by ring expansion [18] to provide 2,3,4,6-tetra–*O*-benzoyl-β-D-galactopyranose-(1→3)-β-D-xylose **29**, which was benzoylated with benzoyl chloride in pyridine. Regioselective removal of the 1-*O*-benzoyl group in 2 M MeOH-NH_3_ followed by trichloroacetimidation with trichloroacetonitrile [9] afforded building block **15 **in 68% yield (3 steps). Finally, condensation of the donor **10 **with the acceptor **9** in the presence of TMSOTf gave benzyl oleanolate 3-*O*-4-*O*-allyl-2,3-di-*O*-benzoyl-α-L-rhamnopyranoside **30 **in 88% yield (Scheme 2). The structure was confirmed by its ^1^H-NMR spectrum, showing characteristic signals at δ 4.97 ppm (*J*_1,2_ = 1.7 Hz) for the H-1 of rhamnose, *δ* 5.81 ppm for CH_2_=CH-CH_2_O, and *δ* 1.12, 1.02, 0.92, 0.92, 0.89, 0.88, 0.61 ppm for CH_3_ of oleanolic acid, the ^13^C-NMR spectrum showed peaks at *δ* 99.6 ppm for anomeric C-1. Deallylation of **30** gave the desired acceptor **31** in 94% yields. The other five oleanolic acid glycosides **34**, **36**, **38**, **40** and **42** were prepared from condensation of the donors and the acceptors **12** and **9**, **13** and **9**, **14** and **16**, **12** and **31**, **15** and **16** respectively, giving 86%~90% yields.

The 28-*O*-benzyl groups in **31**, **16**,**34**, **36**, **38**, **40**, **42** were removed with Pd-C under H_2_ atmosphere, and then the *O*-benzoyl groups were cleaved with MeOH-MeONa [19], furnishing the target compounds **1-7** in satisfactory yields, the structure of the target compounds were established by ^1^H-NMR and ^13^C-NMR spectroscopy. For example, the ^1^H-NMR spectrum of **7 **showed characteristic signals such as *δ* 5.26, 5.25, 4.72 ppm for three H-1, and *δ* 1.28, 1.00, 0.99, 0.95, 0.90, 0.83, 0.78 for the CH_3 _groups of oleanolic acid, the ^13^C-NMR spectrum showed peaks at *δ* 106.3, 105.9, 104.9 ppm for three anomeric C-1s.

### 2.2. Bioassay of Fungicidal Activities

Fungicidal activities of the target compounds against *Sclerotinia sclerotiorum (Lib.) de Bary*, *Rhizoctonia solani Kuhn, Botrytis cinerea Pers* and *Phytophthora parasitica Dast* were evaluated using the mycelium growth rate test [20]. The diameter of the mycelia was measured and the inhibition rate was calculated according to formula (1):

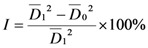
(1)
where *I* is the inhibition rate, 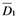 is the average diameter of mycelia in the blank test, and 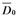 is the average diameter of mycelia in the presence of compounds **1-7**: The inhibition rates of compounds **1-7 **against the four fungi at 50 µg/mL are given in Table 1. Compounds **1-7** exhibited more fungicidal activity against *R. solani* than the other fungi, compounds **1** and **2** are more active against *B. cinerea* and *Phytophthora parasitica Dast* than the other compounds. 

**Table 1:** Inhibition Rate of Compounds **1-7 **against four Fungi.

Compd no.	Inhibition rate (%)				
	*S. sclerotiorum*	*R. solani*	*B. cinerea*	*Phytophthora parasitica Dast*	
1	71.90	96.05	75.41	79.21	
2	67.35	93.24	77.29	83.54	
3	78.27	95.29	68.42	67.24	
4	65.16	96.17	74.59	63.55	
5	73.47	93.86	71.73	52.57	
6	71.90	95.93	71.44	69.72	
7	71.10	88.48	67.17	70.06	

## 3. Experimental

### 3.1. General methods

Solvents were purified in the usual way. All commercially available reagents were used as received. All reactions were monitored by TLC analysis and TLC was performed on silica gel HF with detection by charring with 30% (v/v) H_2_SO_4_ in CH_3_OH or by UV detection. Column chromatography was conducted by elution of a column (8 × 100, 16 × 240, 18 × 300, 35 × 400mm) of silica gel (200-300 mesh) with EtOAc-PE (b.p. 60-90 °C) as the eluent. Air and moisture sensitive reactions were performed under dry N_2 _atmosphere. Optical rotations were recorded using a Perkin-Elmer 241 polarimeter. NMR spectra were recorded on a Varian XL-300 spectrometer with TMS as the internal standard. Elemental analysis was performed on a Yanaco CHN Corder MF-3 automatic elemental analyzer. Mass spectra were recorded with a VG PLATFORM mass spectrometer using the electronspray ionization (ESI) mode. Solutions were concentrated at a temperature <60 °C under diminished pressure.

### 3.2. Chemical synthesis

*p-Methoxyphenyl 4-O-allyl-2,3-O-isopropylidene-α-L-rhamnopyranoside* (**18**). Sodium hydride (2.3 g, 47.4 mmol) and allyl bromide (3.6 mL, 41.1 mmol) were successively added to a soln. of compound **17 [14]** (9.8 g, 31.6 mmol) in N,N-dimethylformamide (50 mL) which was cooled in an ice-salt bath. Then the reaction mixture was slowly allowed to reach room temperature and stirred for 20 min at the end of which time TLC (4:1 petroleum ether-EtOAc) indicated that the reaction was complete. The reaction mixture was diluted with EtOAc (100 mL), washed with ice-water, and dried (Na_2_SO_4_). The soln was concentrated, and the residue was subjected to column chromatography (8:1 petroleum ether-EtOAc) to give the desired product **18** (10.5 g, 95%) as a foamy solid. *R*_f _= 0.68 (4:1 petroleum ether-EtOAc); 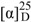-61.4 (*c* 0.5, CHCl_3_); ^1^H-NMR (CDCl_3_): *δ* 7.00-6.81 (m, 4 H, Bz-H), 5.93 (m, 1 H, CH_2_=CH-CH_2_O), 5.59 (s, 1 H, H-1), 5.32-5.16 (m, 2 H), 4.40-4.32 (m, 3 H), 4.14 (m, 1 H), 3.83-3.77 (m, 4 H, H-5, OCH_3_), 3.21 (m, 1 H), 1.56 (s, 3 H, CH_3_), 1.40 (s, 3 H, CH_3_), 1.23 (d, 3 H, *J* = 6.3 Hz, H-6). Anal. Calcd. for C_19_H_26_O_6_: C, 65.13; H, 7.48; found: C, 65.29; H, 7.63.

*p-Methoxyphenyl 4-O-allyl-2,3-di-O-benzoyl-α-L-rhamnopyranoside* (**19**). Compound **18** (7.8 g, 22.3 mmol) was dissolved in 70% HOAc (200 mL) and stirred for 2 h at 75°C, at the end of which time TLC (2:1 petroleum ether-EtOAc) indicated the completion of the reaction. The mixture was concentrated under reduced pressure and then coevaporated with toluene (2 × 40 mL). To a soln of the residue (7.3 g, 23.5 mmol) in pyridine (60 mL) was added benzoyl chloride (8.2 mL, 70.5 mmol) dropwise. After stirring for 8 h at rt, TLC (3:1 petroleum ether-EtOAc) indicated that the reaction was complete. Methanol (1 mL) was added to quench the reaction and then water (100 mL) was added to the reaction mixture. The aq. soln. was extracted with EtOAc (3 × 200 mL), the extract was washed with 1 M HCl and saturated aq. sodium bicarbonate, dried (Na_2_SO_4_) and concentrated. The residue was passed through a short silica-gel column with 6:1 petroleum ether-EtOAc as the eluent to give **19** (10.2 g, 88% for two steps) as a foamy solid. *R*_f _= 0.42 (4:1 petroleum ether-EtOAc); 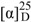 +21.1 (*c* 0.5, CHCl_3_); ^1^H-NMR (CDCl_3_): *δ* 8.07-7.34 (m, 10 H, Bz-H), 7.08-6.83 (m, 4 H, MeOC_6_H_4_), 5.87-5.75 (m, 3 H), 5.52 (d, 1 H, *J* = 1.8 Hz, H-1), 5.17 (m, 1 H), 5.08 (m, 1 H), 4.20-4.08 (m, 3 H), 3.78-3.71 (m, 4 H, H-5, OCH_3_), 1.41 (d, *J* = 6.2 Hz, 3 H, H-6); Anal. Calcd. for C_30_H_30_O_8_: C, 69.49; H, 5.83; found: C, 69.55; H, 5.58;

*4-O-Allyl-2,3-di-O-benzoyl-α-L-rhamnopyranosyl trichloroacetimidate* (**10*)****.* To a soln. of **19** (10.0 g, 19.3 mmol) in 80% MeCN (200 mL) was added ceric ammonium nitrate (42.3 g, 77.2 mmol). The mixture was stirred for 20 min at 35 °C, at the end of which time TLC (4:1 petroleum ether-EtOAc) indicated that the reaction was complete. The solvents were evaporated *in vacuo* at 50 °C to give a residue, which was dissolved in CH_2_Cl_2_, and washed with water. The organic phase was dried (Na_2_SO_4_) and concentrated. Purification by silica gel chromatography with 5:1 petroleum ether-EtOAc as the eluent afforded a foamy residue. The residue was dried under high vacuum for 2 h, then was dissolved in dry CH_2_Cl_2_ (50 mL), trichloroacetonitrile (2.5 mL, 24.3 mmol) and 1,8-diaza-bicyclo[5.4.0] undecene (DBU) (0.3 mL, 30 mmol) were added. The mixture was aged under the nitrogen atmosphere until completion (TLC, 4:1 petroleum ether-EtOAc). Concentration of the reaction mixture and purification of the residue by column chromatography (5:1 petroleum ether-EtOAc) gave **10 **(7.6 g, 72% for two steps) as a white foamy solid. *R*_f _= 0.67 (4:1 petroleum ether-EtOAc); 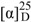 +39.3 (*c* 0.5, CHCl_3_); ^1^H-NMR (CDCl_3_): *δ* 8.74 (s, 1 H, C=NH), 8.06-7.34 (m, 10 H, Bz-H ), 6.39 (d, 1 H, *J* = 1.9 Hz, H-1), 5.85-5.71 (m, 3 H), 5.21-5.07 (m, 2 H), 4.22-4.15 (m, 3 H), 3.77 (dd, 1 H, *J* = 9.6, 9.6 Hz, H-4), 1.48 (d, *J* = 6.2 Hz, 3 H, H-6). Anal. Calcd. for C_25_H_24_Cl_3_NO_7_: C, 53.93; H, 4.34; N, 2.52; found: C, 53.79; H, 4.23; N, 2.29.

*p-Methoxyphenyl 4-O-allyl-2,3-O-isopropylidene-6-deoxy-α-L-talopyranoside* (**21**). Compound **20** (4.9 g, 15.8 mmol) was allylated under the same conditions as used for the preparation of **18** from **17**, giving **21** (5.1 g, 92%) as a foamy solid; *R*_f _= 0.73 (4:1 petroleum ether-EtOAc); 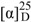 -49.1 (*c* 0.5, CHCl_3_); ^1^H-NMR (CDCl_3_): *δ* 7.01-6.80 (m, 4 H, Bz-H), 5.93 (m, 1 H, CH_2_=CH-CH_2_O), 5.56 (d, 1 H, *J* = 1.5 Hz, H-1), 5.29-5.17 (m, 2 H), 4.49 (m, 1 H), 4.34-4.25 (m, 2 H), 4.09-4.00 (m, 2 H), 3.77 (s, 3 H, OCH_3_), 3.60 (m, 1 H), 1.59 (s, 3 H, CH_3_), 1.40 (s, 3 H, CH_3_), 1.31 (d, 3 H, *J* = 6.6 Hz, H-6). Anal. Calcd. for C_19_H_26_O_6_: C, 65.13; H, 7.48; found: C, 65.25; H, 7.29.

*p-Methoxyphenyl 4-O-allyl-2,3-di-O-benzoyl-6-deoxy-α-L-talopyranoside* (**22**). Sequential de-*O*-isopropylidenation and then benzoylation of compound **21** (7.8 g, 22.3 mmol) under the same conditions as those used for the preparation of **19** from **18**, gave **22** (9.3 g, 80%) as a foamy solid; *R*_f _= 0.67 (3:1 petroleum ether-EtOAc); 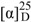 -7.7 (*c* 0.5, CHCl_3_); ^1^H-NMR (CDCl_3_): *δ* 8.25-7.25 (m, 10 H, Bz-H), 7.07-6.82 (m, 4 H, MeOC_6_H_4_), 5.93 (m, 1 H, CH_2_=CH-CH_2_O), 5.77 (dd, 1 H, *J* = 3.49, 3.34 Hz, H-3), 5.68-5.67 (m, 2 H), 5.29-5.12 (m, 2 H), 4.33-4.27 (m, 2 H), 4.07 (m, 1 H), 3.83-3.76 (m, 4 H, CH_2_=CH-CH_2_O, OCH_3_), 1.37 (d, *J* = 6.5 Hz, 3 H, H-6); Anal. Calcd. for C_30_H_30_O_8_: C, 69.49; H, 5.83; found: C, 69.63; H, 5.66.

*4-O-allyl-2,3-di-O-benzoyl-6-deoxy-α-L-talopyranosyl trichloroacetimidate* (**11**). Compound **22** (5.0 g, 9.7 mmol) was trichloroacetimidated under the same conditions as used for the preparation of **10** from **19**, giving **11 **(3.7 g, 70% for two steps) as a foamy solid. *R*_f _= 0.70 (4:1 petroleum ether-EtOAc); 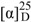 +6.14 (*c* 0.5, CHCl_3_);^ 1^H-NMR (CDCl_3_): *δ* 8.74 (s, 1 H, C=NH), 8.24-7.32 (m, 10 H, Bz-H), 6.48 (d, *J* = 1.4 Hz, 1 H, H-1), 5.90 (m, 1 H), 5.67-5.62 (m, 2 H), 5.18-5.09 (m, 2 H), 4.31-4.07 (m, 2 H), 3.87 (m, 1 H), 3.70 (m, 1 H), 1.44 (d, *J* = 6.5 Hz, 3 H, H-6). Anal. Calcd. for C_25_H_24_Cl_3_NO_7_: C, 53.93; H, 4.34; N, 2.52; found: C, 53.87; H, 4.15; N, 2.78.

*Benzyl oleanolate 3-O-4-O-allyl-2,3-di-O-benzoyl-6-deoxy-α-L-talopyranoside* (**23**). Compound **11** (4.3 g, 7.8 mmol), **9 [10]** (3.6 g, 6.4 mmol) and 4 Å molecular sieves (1.0 g) were added to anhydrous redistilled CH_2_Cl_2_ (60 mL). TMSOTf (130 μL, 0.7 mmol) was added dropwise at -10 °C under nitrogen protection. The reaction mixture was allowed to raise to rt and stirred for 2 h, and then quenched with Et_3_N (2 drops). Filtration of the reaction mixture, concentration of the filtrate, followed by purification of the residue by column chromatography (5:1 petroleum ether-EtOAc) provided **23** (5.2 g, 85%). *R*_f_= 0.47 (8:1 petroleum ether-EtOAc). 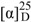 +39.3 (*c* 0.5, CHCl_3_), ^1^H-NMR (CDCl_3_): *δ* 8.23-7.30 (m, 15 H, Ar-H), 5.88 (m, 1 H, CH_2_=CH-CH_2_O), 5.53 (dd, 1 H, *J* = 3.4, 3.5 Hz, H-3'), 5.42 (m, 1 H), 5.29 (br s, 1 H, H-12)**, **5.23-5.02 (m, 5 H), 4.32-4.23 (m, 2 H), 4.03 (m, 1 H), 3.76 (s, 1 H, CH_2_=CH-CH_2_O), 3.17 (dd, 1 H, *J* = 5.1, 10.7 Hz, H-3), 2.90 (dd, 1 H, *J* = 3.8, 13.7 Hz, H-18), 1.35 (d, 3 H, *J* = 6.5 Hz, H-6'), 1.12, 1.01, 0.92, 0.91, 0.89, 0.83, 0.60 (s, 7 × 3 H, CH_3_); ^13^C-NMR (CDCl_3_): *δ* 177.4, 166.3, 165.6 (3 C=O), 143.7, 136.4, 135.0, 133.1, 133.0, 130.3, 130.1, 129.7, 129.7, 128.4, 128.4, 128.4, 128.4, 128.4, 128.2, 128.2, 128.0, 128.0, 128.0, 127.9, 122.5, 116.7, 100.6 (C-1'), 89.3, 76.4, 74.3, 70.1, 69.0, 66.5, 65.9, 55.4, 47.6, 46.7, 45.9, 41.7, 41.4, 39.3, 39.0, 38.4, 36.7, 33.9, 33.1, 32.7, 32.4, 30.7, 28.3, 27.6, 25.8, 25.2, 23.6, 23.4, 23.1, 18.3, 16.9, 16.5, 16.5, 15.3; Anal. Calcd. for C_60_H_76_O_9_: C, 76.56; H, 8.14; found: C, 76.65; H, 8.31.

*Benzyl oleanolate 3-O-2,3-di-O-benzoyl-6-deoxy-α-L-talopyranoside* (**16**). To a soln of compound **23** (5.0 g, 5.2 mmol) in MeOH-CH_2_Cl_2 _= 1/1 (50 mL) was added PdCl_2_ (304 mg, 1.0 mmol). The mixture was stirred for 12 h, at the end of which time TLC (8:1 petroleum ether-EtOAc) indicated that the reaction was complete. The reaction mixture was diluted with dichloromethane (100 mL), washed with water and satd aq Na_2_CO_3_. The organic layer was concentrated, and the residue was passed through a short silica gel column with 8:1 petroleum ether-EtOAc as the eluent to give **16 **(4.4 g, 92%). *R_f_*= 0.32 (8:1 petroleum ether-EtOAc). 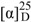 +45.0 (*c* 0.5, CHCl_3_), ^1^H-NMR (CDCl_3_): *δ* 8.07-7.26 (m, 15 H, Ar-H), 5.49-5.47 (m, 2 H), 5.29 (br s, 1 H, H-12)**, **5.07 (m, 3 H), 4.32-3.96 (m, 2 H, H-4', H-5'), 3.20 (dd, 1 H, *J* = 5.8, 9.8 Hz, H-3), 2.90 (dd, 1 H, *J* = 4.4, 13.9 Hz, H-18), 2.55 (d, 1 H, *J* = 11.1 Hz, OH), 1.34 (d, 3 H, *J* = 6.5 Hz, H-6'), 1.12, 1.02, 0.92, 0.92, 0.89, 0.85, 0.61 (s, 7 × 3 H, CH_3_); ^13^C-NMR (CDCl_3_): *δ* 177.4, 165.5, 165.5 (3 C=O), 143.7, 136.5, 133.6, 133.2, 129.8, 129.8, 129.7, 129.7, 129.5, 128.7, 128.7, 128.4, 128.4, 128.3, 128.0, 128.0, 127.9, 127.9, 126.8, 122.5, 100.4 (C-1'), 89.8, 70.6, 70.2, 68.9, 66.7, 65.9, 55.4, 47.6, 46.8, 45.9, 41.7, 41.4, 39.3, 39.0, 38.4, 36.7, 33.8, 33.1, 32.7, 32.4, 30.7, 28.3, 27.6, 25.9, 25.3, 23.6, 23.4, 23.1, 18.3, 16.9, 16.5, 16.2, 15.3; Anal. Calcd. for C_57_H_72_O_9_: C, 75.97; H, 8.05; found: C, 75.83; H, 8.19.

*2,3,4,6-Tetra-O-benzoyl-β-D-galactopyranose-(1→3)-1,2:5,6-di-O-isopropylidene-α-D-glucofuranose***(25)**. Compound **12** [11] (3.87 g, 5.2 mmol) and **24 [16]** (1.24 g, 4.8 mmol) were coupled under the same conditions as that used for the preparation of **23** from **11** and **9**, giving **25 **(3.5 g, 88%) as a foamy solid. *R*_f _= 0.16 (4:1 petroleum ether-EtOAc); 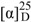 -61.4 (*c* 0.5, CHCl_3_); ^1^H-NMR (CDCl_3_): *δ* 8.09-7.25 (m, 20 H, Bz-H), 5.99 (dd, 1 H, *J* = 0.8, 3.3 Hz), 5.76 (dd, 1 H, *J* = 7.9, 10.5 Hz, H-2'), 5.62 (dd, 1 H, *J* = 3.4, 10.4 Hz, H-3'), 5.50 (d, 1 H, *J* = 3.6 Hz), 4.95 (d, 1 H, *J* = 7.9 Hz, H-1'), 4.67 (dd, 1 H, *J* = 6.3, 11.1 Hz), 4.50-4.25 (m, 6 H), 4.16-4.03 (m, 2 H), 1.43, 1.42, 1.34, 1.12 (s, 4 × 3 H, CH_3_); ^13^C-NMR (CDCl_3_): *δ* 166.0, 165.5, 165.5, 164.9 (4 C=O), 133.6, 133.5, 133.3, 133.3, 129.9, 129.9, 129.9, 129.9, 129.9, 129.8, 129.6, 129.6, 129.4, 129.1, 129.0, 128.7, 128.7, 128.6, 128.6, 128.5, 128.5, 128.3, 128.3, 111.9, 108.6, 104.9, 100.6 (2 × C-1), 82.9, 81.8, 80.5, 77.2, 73.1, 71.8, 71.5, 69.9, 68.0, 66.3, 61.9, 26.7, 26.6, 25.9, 25.3; Anal. Calcd. for C_46_H_46_O_15_: C, 65.86; H, 5.53; found: C, 65.72; H, 5.75.

*2,3,4,6-Tetra-O-benzoyl-β-D-galactopyranose-(1→3)-1,2-O-isopropylidene-α-D-glucofuranose* (**26**). The compound **25** (3.0 g) was dissolved in 60% HOAc (100 mL) and stirred for 6 h at 25°C, at the end of which time TLC (2:1 petroleum ether-EtOAc) indicated the completion of the reaction. The mixture was concentrated under reduced pressure and then co evaporated with toluene (2 × 40 mL). The residue was passed through a short silica-gel column with 3:1 petroleum ether-EtOAc as the eluent to give **26** (2.4 g, 83%) as a foamy solid. *R*_f _= 0.68 (1:1 petroleum ether-EtOAc); 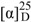 +98.2 (*c* 1.0, CHCl_3_); ^1^H-NMR (CDCl_3_): *δ* 8.08-7.23 (m, 20 H, Bz-H), 6.01 (d, 1 H, *J* = 2.5 Hz), 5.79 (dd, 1 H, *J* = 8.0, 10.5 Hz, H-2'), 5.61 (dd, 1 H, *J* = 3.4, 10.5 Hz, H-3'), 5.53 (d, 1 H, *J* = 3.7 Hz), 4.98 (d, 1 H, *J* = 7.9 Hz, H-1'), 4.57 (d, 2 H, *J* = 6.1 Hz), 4.50-4.29 (m, 3 H), 4.23 (d, 1 H, *J* = 3.7 Hz), 4.19-4.07 (m, 3 H), 3.92-3.85 (m, 1 H), 3.69 (dd, 1 H, *J* = 5.7, 11.5 Hz, H-18), 1.42, 1.06 (s, 2 × 3 H, CH_3_); ^13^C- NMR (CDCl_3_): *δ* 166.1, 165.5, 165.5, 164.8 (4 C=O), 133.8, 133.6, 133.4, 133.4, 133.3, 130.0, 129.9, 129.8, 129.8, 129.8, 129.6, 129.1, 129.0, 129.0, 128.8, 128.8, 128.7, 128.7, 128.6, 128.6, 128.5, 128.3, 128.3, 112.2, 105.2, 101.9 (2 × C-1), 83.6, 83.2, 80.0, 77.2, 72.4, 71.3, 69.5, 68.7, 68.0, 64.4, 62.2, 26.7, 26.2; Anal. Calcd. for C_43_H_42_O_15_: C, 64.66; H, 5.30; found: C, 64.49; H, 5.38.

*2,3,4,6-Tetra-O-benzoyl-β-D-galactopyranose-(1→3)-5-aldehyde-1,2-O-isopropylidene-α-D-gluco-furanose***(27)**. To a vigorously stirred suspension of silicagel-supported NaIO_4_ reagent which was prepared as the reported method [17] (2.0 g) in CH_2_Cl_2_ (5 mL) was added a soln of the compound **26** (0.8 g, 1 mmol) in CH_2_Cl_2_ (5 mL). The mixture was stirred at rt for 25 min, and TLC (2:1 petroleum ether-EtOAc) indicated that the reaction was complete. The mixture was filtered, and the silica gel was thoroughly washed with CHCl_3_. Purification by silica gel chromatography with 2:1 petroleum ether-EtOAc as the eluent afforded **27 **(0.7 g, 88%) as a foamy solid. *R*_f _= 0.41 (2:1 petroleum ether-EtOAc); 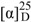 +70.2 (*c* 0.5, CHCl_3_); ^1^H-NMR (CDCl_3_): *δ* 9.68 (d, 1 H, *J* = 1.5 Hz, CHO), 8.08-7.25 (m, 20 H, Bz-H), 5.97 (dd, 1 H, *J* = 0.9, 3.4 Hz), 5.74-5.59 (m, 3 H), 4.89 (d, 1 H, *J* = 7.8 Hz, H-1'), 4.70-4.54 (m, 3 H), 4.48-4.30 (m, 3 H), 1.44, 1.18 (s, 2 × 3 H, CH_3_);^ 13^C-NMR (CDCl_3_): *δ* 197.9, 166.0, 165.7, 165.5, 164.8 (5 C=O), 133.7, 133.6, 133.3, 133.3, 130.3, 130.0, 130.0, 129.9, 129.8, 129.8, 129.8, 129.6, 129.6, 129.4, 129.2, 129.1, 129.0, 128.9, 128.8, 128.7, 128.7, 128.6, 128.5, 128.3, 112.8, 105.7, 100.6 (2 × C-1), 83.9, 83.0, 82.9, 77.2, 71.8, 71.5, 69.6, 67.9, 61.9, 26.6, 26.1; Anal. Calcd. for C_43_H_40_O_14_: C, 66.15; H, 5.16; found: C, 66.34; H, 5.25.

*2,3,4,6-Tetra-O-benzoyl-β-D-galactopyranose-(1→3)-1,2-O-isopropylidene-β-D-xylose* (**28**). To a soln of **27** (1.4 g, 1.8 mmol) in 7:3 EtOAc-H_2_O (50 mL) at 0^ o^C was added NaBH_4_ (109 mg, 2.7 mmol). The mixture was stirred at 0°C for 15 min, and TLC (3:1 petroleum ether-EtOAc) indicated that the reaction was complete. The aq. soln. was extracted with EtOAc (3 × 100 mL), the extract was washed with 1 M HCl and saturated aq sodium bicarbonate, dried (Na_2_SO_4_) and concentrated. Purification by silica gel chromatography with 5:1 petroleum ether-EtOAc as the eluent afforded **28 **(1.3 g, 96%) as a foamy solid. *R*_f _= 0.29 (3:2 petroleum ether-EtOAc); 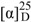 +184.2 (*c* 1.0, CHCl_3_); ^1^H-NMR (CDCl_3_): *δ* 8.08-7.26 (m, 20 H, Bz-H), 6.00 (dd, 1 H, *J* = 0.8, 3.3 Hz), 5.80-5.55 (m, 3 H), 4.96 (d, 1 H, *J* = 7.9 Hz, H-1'), 4.70-4.30 (m, 6 H), 4.16-3.91 (m, 2 H), 2.56 (dd, 1 H, *J* = 6.7, 6.7 Hz), 1.44, 1.12 (s, 2 × 3 H, CH_3_);^ 13^C-NMR (CDCl_3_): *δ* 166.1, 165.5, 165.5, 164.9 (4 C=O), 133.8, 133.6, 133.4, , 133.4, 130.1, 130.1, 130.0, 130.0, 129.8, 129.8, 129.7, 129.6, 129.2, 129.0, 128.9, 128.8, 128.7, 128.7, 128.7, 128.6, 128.5, 128.5, 128.3, 112.1, 104.9, 101.3 (2 × C-1), 83.6, 82.8, 79.8, 77.2, 72.1, 71.4, 69.6, 68.0, 62.2, 59.9, 26.9, 26.1; Anal. Calcd. for C_42_H_40_O_14_: C, 65.62; H, 5.24; found: C, 65.35; H, 5.37.

*2,3,4,6-Tetra-O-benzoyl-β-D-galactopyranose-(1→3)-β-D-xylose* (**29**). Compound **28** (1.22 g, 1.6 mmol) was dissolved in 4% aq H_2_SO_4 _(100 mL) and then refluxed for 4 h. TLC (1:1 petroleum ether-EtOAc) indicated that the reaction was complete. The resulting soln. was cooled down to room temperature and extracted three times with EtOAc. The extract was washed with saturated aq. sodium bicarbonate, dried (Na_2_SO_4_) and concentrated. Purification by silica gel chromatography with 2:1 petroleum ether-EtOAc as the eluent afforded **29 **(0.9 g, 85%) as a foamy solid. *R*_f _= 0.31 (1:1 petroleum ether-EtOAc); 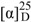 +331.6 (c 1.0, CHCl_3_); ^1^H-NMR (CDCl_3_): *δ* 8.10-7.25 (m, 20 H, Bz-H), 6.01 (d, 1 H, *J* = 3.3 Hz**), **5.85 (m, 1 H), 5.65 (m, 1 H), 5.07-5.02 (m, 2 H), 4.58-4.40 (m, 3 H), 3.79-3.74 (m, 3 H), 3.50 (m, 1 H), 3.27 (m, 1 H);^ 13^C-NMR (CDCl_3_): *δ* 166.1, 165.6, 165.5, 165.5 (4 C=O), 133.7, 133.5, 133.4, 133.4, 130.0, 130.0, 129.8, 129.7, 129.1, 129.0, 128.8, 128.8, 128.7, 128.6, 128.5, 128.5, 128.4, 128.3, 102.8, 102.6 (2 × C-1), 97.3, 92.4, 88.2, 85.7, 77.2, 73.4, 72.0, 71.5, 70.7, 70.0, 69.9, 68.2, 68.1, 62.4, 62.0; Anal. Calcd. for C_39_H_36_O_14_: C, 64.28; H, 4.98; found: C, 64.39; H, 4.83.

*2,3,4,6-Tetra-O-benzoyl-β-D-galactopyranose-(1→3)-2,4-di-O-benzoyl-β-D-xylopyranosyl trichloro-acetimidate* (**15**). Compound **29** (3.5 g, 4.8 mmol) was benzoylated under the same conditions as used for the preparation of **19**. Then the resultant residue was dissolved in 2 M MeOH-NH_3_ (200 mL) and stirred at 35 °C at the end of which time TLC (3:1 petroleum ether-EtOAc) indicated that the reaction was complete. The solvents were evaporated *in vacuo* at 50 °C to give a residue, which was dissolved in CH_2_Cl_2_, and washed with water. The organic phase was dried (Na_2_SO_4_) and concentrated. Purification by silica gel chromatography with 5:1 petroleum ether-EtOAc as the eluent afforded a foamy residue. The residue was trichloroacetimidated under the same conditions as used for the preparation of **10** from **19**, giving **15 **(3.5 g, 68% for three steps) as a white foamy solid. *R*_f _= 0.42 (3:1 petroleum ether-EtOAc); 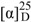 +18.4 (*c* 0.5, CHCl_3_); ^1^H-NMR (CDCl_3_): *δ* 8.52 (s, 1 H, C=NH), 8.18-7.08 (m, 30 H, Bz-H ), 6.54 (d, 1 H, *J* = 3.5 Hz), 5.87 (d, 1 H, *J* = 3.3 Hz), 5.66 (dd, 1 H, *J* = 7.9, 10.4 Hz), 5.48-5.37 (m, 2 H), 5.27 (dd, 1 H, *J* = 3.5, 9.7 Hz), 5.14 (d, 1 H, *J* = 7.9 Hz, H-1'), 4.67 (dd, 1 H, *J* = 9.4, 9.4 Hz), 4.41-4.18 (m, 4 H), 3.95 (dd, 1 H, *J* = 10.9, 11.0 Hz). Anal. Calcd. for C_55_H_44_Cl_3_NO_16_: C, 61.09; H, 4.10; N, 1.30; found: C, 61.34; H, 4.27; N, 1.49.

*Benzyl oleanolate 3-O-4-O-allyl-2,3-di-O-benzoyl-α-L-rhamnopyranoside* (**30**). Compound **10** (4.3 g, 7.8 mmol) and **9 [10]** (3.6 g, 6.4 mmol) were coupled under the same conditions as used for the preparation of **23** from **11** and **9**, giving **30 **(5.4 g, 88%) as a foamy solid. *R*_f _= 0.45 (8:1 petroleum ether-EtOAc); 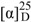 +73.7 (*c* 0.5, CHCl_3_); ^1^H-NMR (CDCl_3_): *δ* 8.06-7.31 (m, 15 H, Ar-H), 5.81 (m, 1 H, CH_2_=CH-CH_2_O), 5.66 (dd, 1 H, *J* = 3.2, 9.5 Hz, H-3'), 5.57 (dd, 1 H, *J* = 1.7, 3.2 Hz, H-2'), 5.29 (br s, 1 H, H-12)**, **5.20-5.03 (m, 4 H, PhCH2, CH_2_=CH-CH_2_O), 4.97 (d, 1 H, *J*_1,2 _= 1.7 Hz, H-1'), 4.22-4.04 (m, 3 H), 3.67 (dd, 1 H, *J* = 9.5, 9.5 Hz, H-4'), 3.15 (dd, 1 H, *J* = 6.3, 9.8 Hz, H-3), 2.91 (dd, 1 H, *J* = 4.1, 13.4 Hz, H-18), 1.40 (d, 3 H, *J* = 6.2 Hz, H-6'), 1.12, 1.02, 0.92, 0.92, 0.89, 0.88, 0.61 (s, 7 × 3H, CH_3_); ^13^C-NMR (CDCl_3_): *δ* 177.4, 165.5, 165.3 (3 C=O), 143.7, 136.4, 134.5, 133.2, 132.9, 130.0, 130.0, 129.9, 129.7, 129.5, 129.5, 128.4, 128.4, 128.4, 128.4, 128.3, 128.0, 128.0, 127.9, 122.5, 117.2, 99.6 (C-1'), 89.7, 79.1, 77.2, 74.0, 72.5, 71.5, 67.8, 65.9, 55.4, 47.6, 45.9, 41.7, 41.4, 39.3, 39.0, 38.4, 36.7, 33.9, 33.1, 32.7, 32.4, 30.7, 28.3, 27.6, 25.9, 25.3, 23.6, 23.4, 23.1, 18.3, 18.0, 16.9, 16.5, 15.3; Anal. Calcd. for C_60_H_76_O_9_: C, 76.56; H, 8.14; found: C, 76.73; H, 8.43.

*Benzyl oleanolate 3-O-2,3-di-O-benzoyl-α-L-rhamnopyranoside* (**31**). Compound **30** (5.0 g, 5.2 mmol) was deallylated under the same conditions as that used for the preparation of **16 **from **23**, giving **31** (4.5 g, 94%) as a foamy solid; *R*_f _= 0.16 (8:1 petroleum ether-EtOAc); 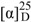 +29.5 (*c* 1.0, CHCl_3_); ^1^H- NMR (CDCl_3_): *δ* 8.09-7.26 (m, 15 H, Ar-H), 5.57-5.48 (m, 2 H), 5.29 (br s, 1 H, H-12)**, **5.07 (dd, 2 H, *J* = 12.6, 17.1 Hz, PhCH_2_), 5.00 (d, 1 H, *J* = 1.6 Hz, H-1'), 4.06-3.87 (m, 2 H), 3.18 (dd, 1 H, *J* = 2.9, 13.0 Hz, H-3), 2.90 (dd, 1 H, *J* = 4.4, 14.2 Hz, H-18), 2.49 (d, 1 H, *J* = 5.1 Hz, OH), 1.41 (d, 3 H, *J* = 6.1 Hz, H-6'), 1.12, 1.01, 0.92, 0.92, 0.89, 0.87, 0.61 (s, 7 × 3 H, CH_3_); ^13^C-NMR (CDCl_3_): *δ* 177.4, 166.8, 165.6 (3 C=O), 143.6, 136.4, 133.3, 133.2, 129.7, 129.7, 129.7, 129.7, 129.5, 129.4, 128.4, 128.4, 128.3, 128.3, 127.9, 127.9, 127.9, 127.8, 126.8, 122.4, 99.7 (C-1'), 89.7, 73.4, 72.1, 71.3, 68.7, 65.9, 55.4, 47.5, 46.7, 45.8, 41.6, 41.4, 39.3, 38.9, 38.4, 36.7, 33.8, 33.0, 32.7, 32.3, 30.6, 28.3, 27.6, 25.8, 25.3, 23.6, 23.4, 23.0, 18.2, 17.5, 16.8, 16.5, 15.3; Anal. Calcd. for C_57_H_72_O_9_: C, 75.97; H, 8.05; found: C, 75.81; H, 8.29.

*Oleanolic acid 3-O-α-L-rhamnopyranoside* (**1**). A suspension of **31** (1.3 g, 1.4 mmol) and 10% Pd-C (1.5 g) in EtOAc (30 mL) was refluxed and bubbled up with H_2 _(20 mL/min). When TLC (2:1, petroleum-EtOAc) showed that the reaction had completed, Pd-C was removed through filtration and the filtrate was concentrated to dryness. The resulted amorphous solid was dissolved in dry CH_2_Cl_2_-MeOH (1:2, 30 mL), to which a newly prepared NaOMe/MeOH (1.0 mol/L, 20 mL) was added. The soln was stirred at rt for 2 h and then neutralized with Dowex H^+^ resin to pH 7 and filtered. The filtrate was concentrated and subjected to a flash column chromatography (CHCl_3_-MeOH-H_2_O 7:3:1, organic layer) to give **1 [13]** (737 mg, 86% for two steps) as a white powder. 

*Oleanolic acid 3-O-6-deoxy-α-L-talopyranoside* (**2**). Compound **2** was prepared from **16** by the same procedure as for **1**. Yield: 81%; white powder, m.p. 288-290 °C, *R*_f _= 0.29 (10:1:0.1 CHCl_3_-MeOH-H_2_O); 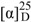 +6.1 (*c* 0.5, MeOH); ^1^H-NMR (pyridine-d_5_): *δ* 5.47 (br s, 1 H, H-12)**, **5.31 (d, 1 H, *J* = 1.3 Hz, H-1'), 4.85 (dd, 1 H, *J* = 1.5, 3.0 Hz, H-2'), 4.25-4.21 (m, 2 H), 4.06 (d, 1 H, *J* = 1.4 Hz), 3.29 (dd, 1 H, *J* = 4.0, 13.7 Hz, H-3), 3.13 (dd, 1 H, *J* = 4.3, 11.5 Hz, H-18), 1.54 (d, 3 H, *J* = 6.5 Hz, H-6'), 1.28, 1.00, 0.99, 0.95, 0.90, 0.85, 0.80 (s, 7 × 3 H, CH_3_); ^13^C-NMR (pyridine-d_5_): *δ* 180.2, 144.9, 122.6, 104.9 (C-1'), 88.6, 74.3, 72.4, 67.8, 67.5, 55.7, 48.1, 46.7, 46.6, 42.2, 42.1, 39.8, 39.2, 38.6, 37.1, 34.3, 33.4, 33.3, 33.2, 31.0, 28.4, 28.3, 26.2, 25.8, 23.9, 23.9, 23.8, 18.7, 17.5, 17.4, 16.8, 15.5; HRESIMS: *m/z* calcd. for C_36_H_58_O_7_Na[M+Na^+^]: 625.4080; found: *m/z* 625.4059.

*Benzyl oleanolate 3-O-2,3,4,6-tetra-O-benzoyl-β-D-galactopyranoside* (**34**). Compound **12** (0.56 g, 0.8 mmol) and **9** [15] (0.6 g, 0.7 mmol) were coupled under the same conditions as that used for the preparation of **23** from **11** and **9**, giving **34 [13]** (0.9 g, 90%) as a foamy solid.

*Oleanolic acid 3-O-β-D-galactopyranoside* (**3**). Compound **3 [13]** was prepared from **34** by the same procedure as for **1**. Yield: 84%; white powder.

*Benzyl oleanolate 3-O-2,3,4,6-tetra-O-acetyl-α-D-mannopyranoside* (**36**). Compound **13** [12] (1.5 g, 3.0 mmol) and **9 [10]** (1.4 g, 2.5 mmol) were coupled under the same conditions as used for the preparation of **23** from **11** and **9**, giving **36 **(1.9 g, 86%) as a foamy solid. *R*_f _= 0.16 (6:1 petroleum ether-EtOAc); 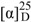 +70.6 (*c* 0.5, CHCl_3_); ^1^H-NMR (CDCl_3_): *δ* 7.47-7.27 (m, 5 H, Bn-H), 5.35-5.24 (m, 3 H), 5.16-5.06 (m, 3 H), 4.97 (d, 1 H, *J* = 1.7 Hz, H-1'), 4.25 (dd, 1 H, *J* = 5.7, 12.5 Hz, H-3), 4.15-4.10 (m, 2 H), 3.21 (dd, 1 H, *J* = 4.0, 11.3 Hz, H-3), 2.90 (dd, 1 H, *J* = 4.0, 13.6 Hz, H-18), 2.16, 2.09, 2.05, 2.00 (s, 4 × 3 H, CH_3_CO), 1.11, 1.00, 0.92, 0.89, 0.89, 0.82, 0.60 (s, 7 × 3 H, CH_3_); ^13^C- NMR (CDCl_3_): *δ* 177.4, 170.6, 170.2, 169.9, 169.8 (5 C=O), 143.6, 136.3, 128.3, 128.3, 127.9, 127.9, 122.4, 94.6 (C-1'), 84.7, 77.2, 70.7, 69.2, 69.0, 66.4, 66.3, 65.9, 62.6, 55.6, 47.6, 46.7, 45.8, 41.6, 41.3, 39.3, 38.3, 38.0, 36.8, 33.8, 33.0, 32.7, 32.3, 30.6, 28.7, 27.6, 25.8, 23.6, 23.4, 23.0, 22.1, 20.8, 20.6, 20.6, 18.2, 16.8, 16.4, 15.2; HRESIMS: *m/z* calcd. for C_51_H_72_O_8_Na[M+Na^+^]: 835.5125; found: *m/z* 835.5118.

*Oleanolic acid 3-O-α-D-mannopyranoside* (**4**). Compound **4** was prepared from **36** by the same procedure as for **1**. Yield: 87%; white powder, m.p. 250-252 °C, *R*_f _= 0.07 (20:1:0.1 CHCl_3_-MeOH-H_2_O); 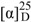 +79.8 (*c* 0.5, MeOH); ^1^H-NMR (pyridine-d_5_): *δ* 5.54 (d, 1 H, *J* = 1.0 Hz, H-1'), 5.46 (br s, 1 H, H-12), 4.69 (m, 1 H), 4.59-4.50 (m, 3 H), 4.46-4.38 (m, 2 H), 3.47 (dd, 1 H, *J* = 4.2, 11.4 Hz, H-3), 3.28 (dd, 1 H, *J* = 4.0, 13.5 Hz, H-18), 1.24, 1.15, 1.00, 0.97, 0.94, 0.81, 0.79 (s, 7 × 3 H, CH_3_); ^13^C-NMR (pyridine-d_5_): *δ* 180.1 (C=O), 144.8, 124.1, 122.4, 97.7 (C-1'), 81.8, 75.8, 73.2, 72.9, 69.2, 63.4, 55.7, 47.9, 46.6, 46.4, 42.1, 41.9, 39.7, 38.5, 38.1, 37.1, 34.2, 33.2, 33.1, 33.1, 30.9, 29.0, 28.2, 26.1, 23.7, 23.6, 22.0, 18.5, 17.3, 16.9, 15.3; HRESIMS: *m/z* calcd. for C_36_H_58_O_8_Na[M+Na^+^]: 641.4029; found: *m/z* 641.4037.

*Benzyl oleanolate 3-O-2,3,4-tri-O-benzoyl-β-D-xylopyranosyl-(1→4)-2,3-di-O-benzoyl-6-deoxy-α-L-talopyranoside* (**38**). Compound **16** (1.1 g, 1.3 mmol) and **14 [13]** (0.9 g, 1.5 mmol) were coupled under the same conditions as that used for the preparation of **23** from **11** and **9**, giving **38 **(1.4 g, 90%) as a foamy solid. *R*_f _= 0.13 (8:1 petroleum ether-EtOAc); 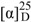 -24.6 (*c* 0.5, CHCl_3_); ^1^H-NMR (CDCl_3_): *δ* 8.33-7.26 (m, 30 H, Ar-H), 5.67 (dd, 1 H, *J* = 8.2, 9.1 Hz, H-2''), 5.58 (dd, 1 H, *J* = 6.7, 9.1 Hz, H-3''), 5.48 (d, 1 H, *J* = 2.2 Hz, H-1'), 5.40 (dd, 1 H, *J* = 3.6, 3.6 Hz, H-3'), 5.28 (br s, 1 H, H-12), 5.07 (dd, 2 H, *J* = 12.5, 17.6 Hz, PhCH_2_), 4.98-4.92 (m, 2 H), 4.70 (d, 1 H, *J* = 6.7 Hz, H-1''), 4.29-4.19 (m, 2 H), 3.50 (m, 1 H), 3.14-3.04 (m, 2 H), 2.89 (dd, 1 H, *J* = 4.1, 9.6 Hz, H-18), 1.19 (d, 3 H, *J* = 6.5 Hz, H-6'), 1.10, 0.97, 0.91, 0.89, 0.89, 0.81, 0.59 (s, 7 × 3 H, CH_3_); ^13^C-NMR (CDCl_3_): *δ* 177.4, 166.3, 166.2, 165.6, 165.2, 164.9 (6 C=O), 143.7, 140.9, 138.8, 136.4, 133.3, 133.2, 133.1, 133.0, 130.4, 130.0, 130.0, 129.9, 129.8, 129.7, 129.2, 129.1, 128.6, 128.5, 128.4, 128.4, 128.3, 128.3, 128.3, 128.0, 127.9, 127.8, 127.6, 127.5, 126.9, 122.4, 103.1, 100.7 (2 × C-1), 99.4, 89.5, 78.1, 77.3, 76.6, 76.4, 74.2, 73.7, 72.8, 72.3, 71.8, 71.6, 69.9, 68.6, 68.3, 65.9, 65.6, 65.3, 62.1, 60.2, 55.5, 55.4, 47.5, 46.7, 45.9, 41.7, 41.4, 39.3, 38.9, 38.4, 36.7, 33.1, 32.4, 30.7, 28.3, 27.6, 25.8, 23.6, 23.4, 23.1, 18.2, 16.9, 16.5, 16.1, 15.3; HRESIMS: *m/z* calcd. for C_83_H_92_O_16_Na[M+Na^+^]: 1367.6283; found: *m/z* 1367.6290.

*Oleanolic acid 3-O-β-D-xylopyranosyl-(1→4)-6-deoxy-α-L-talopyranoside* (**5**). Compound **5** was prepared from **38** by the same procedure as for **1**. Yield: 71%; white powder, m.p. 218-220 °C, *R*_f _= 0.11 (10:1:0.1 CHCl_3_-MeOH-H_2_O); 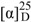 -30.7 (*c* 0.5, MeOH); ^1^H-NMR (pyridine-d_5_): *δ* 5.47 (br s, 1 H, H-12), 5.27 (s, 1 H, H-1'), 4.80 (d, 1 H, *J* = 7.4 Hz, H-1''), 4.34-4.30 (m, 2 H), 4.25-4.20 (m, 3 H), 4.13-3.93 (m, 3 H), 3.69 (dd, 1 H, *J* = 9.6, 10.9 Hz), 3.29 (dd, 1 H, *J* = 3.9, 13.6 Hz, H-3), 3.11 (dd, 1 H, *J* = 4.3, 11.6 Hz, H-18), 1.70 (d, 3 H, *J* = 6.6 Hz, H-6'), 1.29, 1.00, 1.00, 0.95, 0.92, 0.84, 0.79 (s, 7 × 3 H, CH_3_); ^13^C-NMR (pyridine-d_5_): *δ* 180.1 (C=O), 144.8, 122.4, 106.3, 104.9 (2 × C-1), 88.6, 83.3, 77.8, 74.7, 71.9, 70.5, 67.2, 67.1, 66.7, 55.5, 47.9, 46.6, 46.4, 42.1, 42.0, 39.7, 39.1, 38.4, 36.9, 34.2, 33.2, 33.2, 33.1, 30.9, 28.2, 26.1, 25.6, 23.7, 23.7, 23.7, 23.6, 18.5, 17.3, 17.0, 16.6, 15.4; HRESIMS: *m/z* calcd. for C_41_H_66_O_11_Na[M+Na^+^]: 757.4503; found: *m/z* 757.4515.

*Benzyl oleanolate 3-O-2,3,4,6-tetra-O-benzoyl-β-D-galactopyranosyl-(1→4)-2,3-di-O-benzoyl-α-L-rhamnopyranoside* (**40**). Compound **12 [11]** (0.9 g, 1.2 mmol) and **31** (0.9 g, 1.0 mmol) were coupled under the same conditions as that used for the preparation of **23** from **11** and **9**, giving **40 **(1.3 g, 89%) as a foamy solid. *R*_f _= 0.07 (8:1 petroleum ether-EtOAc); 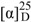 +64.5 (*c* 0.5, CHCl_3_); ^1^H-NMR (CDCl_3_): *δ* 8.07-7.05 (m, 35 H, Ar-H), 5.97 (d, 1 H, *J* = 3.1 Hz, H-4''), 5.75 (dd, 1 H, *J* = 7.9, 10.4 Hz, H-2''), 5.55-5.47 (m, 2 H, H-2', H-3'), 5.41 (dd, 1 H, *J* = 3.3, 10.4 Hz, H-3''), 5.30 (br s, 1 H, H-12), 5.13-5.07 (m, 3 H, H-1'', PhCH_2_), 4.94 (d, 1 H, *J* = 1.4 Hz, H-1'), 4.71-4.37 (m, 3 H), 4.21-4.05 (m, 2 H), 3.14 (dd, 1 H, *J* = 7.5, 8.6 Hz, H-3), 2.91 (dd, 1 H, *J* = 3.9, 13.7 Hz, H-18), 1.50 (d, 1 H, *J* = 6.0 Hz, H-6'), 1.12, 0.96, 0.92, 0.92, 0.89, 0.85, 0.61 (s, 7 × 3 H, CH_3_); ^13^C-NMR (CDCl_3_): *δ* 177.4, 166.1, 165.5, 165.4, 165.4, 165.3, 164.7 (7 C=O), 143.7, 136.4, 133.5, 133.3, 133.2, 133.1, 132.8, 129.9, 129.7, 129.7, 129.7, 129.6, 129.6, 129.6, 129.6, 129.6, 129.5, 129.5, 129.4, 129.3, 129.0, 128.8, 128.6, 128.6, 128.5, 128.5, 128.4, 128.4, 128.4, 128.4, 128.4, 128.4, 128.4, 128.4, 128.3, 128.2, 128.1, 128.0, 128.0, 128.0, 128.0, 127.9, 122.5, 101.5, 99.8 (2 × C-1), 89.9, 77.6, 77.2, 72.5, 71.9, 71.1, 70.9, 69.7, 68.0, 67.1, 65.9, 62.0, 55.4, 47.6, 46.7, 45.9, 41.7, 41.4, 39.3, 38.9, 38.5, 36.7, 33.8, 33.0, 32.7, 32.4, 30.6, 28.3, 27.6, 25.8, 25.4, 23.6, 23.4, 23.1, 18.2, 18.1, 16.8, 16.5, 15.3; HRESIMS: *m/z* calcd. for C_91_H_98_O_18_Na[M+Na^+^]: 1501.6651; found: *m/z* 1501.6629.

*Oleanolic acid 3-O-β-D-galactopyranosyl-(1→4)-α-L-rhamnopyranoside* (**6**). Compound **6** was prepared from **40** by the same procedure as for **1**. Yield: 68%; white powder, m.p. 268-270 °C, *R*_f _= 0.70 (10:2:0.1 CHCl_3_-MeOH-H_2_O); 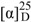 +24.6 (*c* 0.5, MeOH); ^1^H-NMR (pyridine-d_5_): *δ* 5.46 (br s, 1 H, H-12), 5.27 (s, 1 H, H-1'), 5.18 (d, 1 H, *J* = 7.8 Hz, H-1''), 4.60-4.24 (m, 8 H), 4.14 (dd, 1 H, *J* = 3.4, 9.5 Hz), 3.95 (dd, 1 H, *J* = 6.1, 6.4 Hz, H-2''), 3.29 (dd, 1 H, *J* = 4.2, 13.7 Hz, H-3), 3.11 (dd, 1 H, *J* = 4.5, 11.6 Hz, H-18),1.74 (d, 3 H, *J* = 6.2 Hz, H-6'), 1.27, 1.00, 0.99, 0.95, 0.89, 0.83, 0.76 (s, 7 × 3 H, CH_3_); ^13^C-NMR (pyridine-d_5_): *δ* 180.1 (C=O), 144.8, 122.7, 122.5, 107.3, 103.9 (2 × C-1), 88.5, 85.2, 76.9, 75.6, 74.1, 72.9, 71.9, 70.0, 68.1, 62.0, 55.5, 47.9, 46.6, 46.4, 42.1, 42.0, 39.7, 39.1, 38.4, 36.9, 34.2, 33.2, 33.2, 33.1, 30.9, 28.3, 28.2, 26.1, 25.7, 23.7, 23.7, 18.5, 18.3, 17.3, 16.7, 15.4; HRESIMS: *m/z* calcd. for C_42_H_68_O_12_Na[M+Na^+^]: 787.4608; found: *m/z* 787.4579. 

*Benzyl oleanolate 3-O-2,3,4,6-tetra-O-benzoyl-β-D-galactopyranosyl-(1→3)-2,4-di-O-benzoyl-β-D-xylopyranosyl-(1→4)-2,3-di-O-benzoyl-6-deoxy-α-L-talopyranoside* (**42**). Compound **16** (0.3 g, 0.3 mmol) and **15** (0.41 g, 0.4 mmol) were coupled under the same conditions as used for the preparation of **23** from **11** and **9**, giving **42 **(0.5 g, 90%) as a foamy solid. *R*_f _= 0.11 (4:1 petroleum ether-EtOAc); 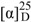 -26.6 (*c* 0.5, CHCl_3_); ^1^H-NMR (CDCl_3_): *δ* 8.28-7.16 (m, 45 H, Ar-H), 5.78 (d, 1 H, *J* = 3.5 Hz), 5.59 (dd, 1 H, *J* = 7.9, 10.4 Hz), 5.47-5.41 (m, 2 H), 5.26-5.20 (m, 2 H), 5.06 (dd, 2 H, *J* = 12.5, 17.3 Hz, PhCH_2_), 4.93-4.86 (m, 3 H), 4.44 (d, 1 H, *J* = 1.4 Hz), 4.39-3.92 (m, 6 H), 3.30 (dd, 1 H, *J* = 5.6, 12.1 Hz, H-3), 3.06-2.87 (m, 3 H), 0.99 (d, 1 H, *J* = 6.5 Hz, H-6'), 1.09, 0.93, 0.91, 0.89, 0.86, 0.77, 0.58 (s, 7 × 3 H, CH_3_); ^13^C-NMR (CDCl_3_) *δ* 177.4, 166.3, 166.2, 165.8, 165.4, 165.3, 165.0, 164.9, 163.9 (9 C=O), 143.7, 136.4, 133.3, 133.3, 133.3, 133.2, 133.2, 133.1, 133.0, 132.9, 130.5, 130.0, 129.9, 129.9, 129.8, 129.8, 129.7, 129.7, 129.7, 129.7, 129.7, 129.7, 129.6, 129.6, 129.6, 129.5, 129.5, 129.4, 129.4, 129.4, 129.4, 129.2, 129.0, 128.7, 128.6, 128.5, 128.5, 128.5, 128.5, 128.5, 128.4, 128.4, 128.4, 128.4, 128.4, 128.4, 128.3, 128.3, 128.2, 128.2, 128.1, 128.0, 127.9, 127.9, 127.8, 127.8, 122.4, 103.3, 101.0, 100.6 (3 × C-1), 89.3, 77.3, 73.2, 71.7, 70.9, 70.2, 69.7, 68.5, 68.0, 67.5, 65.9, 65.4, 62.4, 61.2, 55.3, 47.5, 46.7, 45.9, 41.7, 41.4, 39.3, 38.9, 38.3, 36.6, 33.8, 33.0, 32.6, 32.3, 30.6, 28.2, 27.6, 25.8, 25.1, 23.6, 23.3, 23.0, 18.2, 16.8, 16.4, 15.9, 15.3; HRESIMS: *m/z* calcd. for C_110_H_114_O_24_Na[M+Na^+^]: 1841.7598; found: *m/z* 1841.7579.

*Oleanolic acid 3-O-β-D-galactopyranosyl-(1→3)-β-D-xylopyranosyl-(1→4)-6-deoxy-α-L-talo-pyranoside* (**7**). Compound **7** was prepared from **42** by the same procedure as for **1**. Yield: 75%; white powder, m.p. 202-204 °C, *R*_f _= 0.82 (10:2:0.1 CHCl_3_-MeOH-H_2_O); 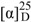 -36.8 (*c* 0.5, MeOH); ^1^H- NMR (pyridine-d_5_): *δ* 5.47 (br s, 1 H, H-12), 5.27-5.25 (m, 2 H), 4.72 (d, 1 H, *J* = 7.8 Hz), 4.54-4.50 (m, 2 H), 4.40-3.91 (m, 12 H), 3.85 (dd, 1 H, *J* = 7.0, 7.0 Hz), 3.59 (dd, 1 H, *J* = 10.2, 11.2 Hz), 3.28 (dd, 1 H, *J* = 4.2, 10.0 Hz, H-3), 3.09 (dd, 1 H, *J* = 4.4, 11.4 Hz, H-18), 1.67 (d, 3 H, *J* = 6.5 Hz, H-6'), 1.28, 1.00, 0.99, 0.95, 0.90, 0.83, 0.78 (s, 7 × 3 H, CH_3_); ^13^C-NMR (pyridine-d_5_): *δ* 180.2, 144.9, 122.5, 106.3, 105.9, 104.9 (3 × C-1), 88.8, 86.8, 83.6, 77.3, 75.2, 73.5, 73.1, 72.0, 70.2, 69.0, 67.1, 66.7, 66.5, 62.1, 57.4, 55.6, 48.0, 46.7, 46.5, 42.2, 42.1, 39.8, 39.2, 38.5, 37.0, 34.3, 33.3, 33.2, 31.0, 28.4, 28.3, 26.2, 25.7, 23.8, 23.7, 19.2, 18.6, 17.4, 17.0, 16.8, 15.5; HRESIMS: *m/z* calcd. for C_47_H_76_O_16_Na[M+Na^+^]: 919.5031; found: *m/z* 919.5018.

### 3.3. Fungicidal activity bioassay

We used the mycelium growth rate test [20]. The culture media, with known concentration of the test compounds, were obtained by mixing the soln of compounds **1-7** in methanol with potato dextrose agar (PDA), on which fungus cakes were placed. The blank test was made using methanol. The culture was carried out at 24 ± 0.5 °C. Three replicates were performed.

## 4. Conclusions

Seven glycoconjugates of oleanolic acid were designed and efficiently synthesized. The bioassays showed that they had some fungicidal activity against four fungi. All of the compounds exhibited more fungicidal activity against *R. solani*, and the compounds **1** and **2** had better activity against *B. cinerea* and *P. CapasiciLeonian* than the other compounds.
